# ENGAGING RURAL OLDER ADULT COMMUNITY CENTERS TO SUPPORT COGNITIVE HEALTH: CBPR LESSONS AND BARRIERS

**DOI:** 10.1093/geroni/igae098.4172

**Published:** 2024-12-31

**Authors:** Megan McCoy, Eric Cerino, Michael McCarthy, Margarita Martinez, Louis Lucero, Thomasina Seaton, Amanda Goldtooth, Raechel Livingston

**Affiliations:** Northern Arizona University, Northern Arizona University, Arizona, United States; Northern Arizona University, Flagstaff, Arizona, United States; Northern Arizona University, Flagstaff, Arizona, United States; Northern Arizona University, Northern Arizona University, Arizona, United States; Joe C. Montoya Community and Senior Center, Flagstaff, Arizona, United States; Northern Arizona University, Flagstaff, Arizona, United States; Northern Arizona University, Flagstaff, Arizona, United States; Northern Arizona University, Flagstaff, Arizona, United States

## Abstract

Academic-community partnerships grounded in community-based participatory research (CBPR) methods can further health equity and improve cognitive health for older adults and caregivers in rural communities. Older adult community centers, specifically, can serve as partners to advance preventative memory screening and early interventions to address cognitive health disparities and provide support for caregivers. The aim of this study is to describe a model for researchers in the early stages of partnership development, aspiring to use CBPR, to better align activities with community needs. Informed by the Quality Improvement and Innovation Partnership (QIIP) model, we describe the process of engaging older adult community centers as research partners to advance the Northern Arizona Memory Study. Descriptive findings from surveys with 6 center leaders, combined with thematic analysis of partnership development process notes, illuminate lessons and barriers to engagement, as well as conceptual advances for CBPR methods. Lessons include the importance of honoring mutual expertise, informal engagement with center staff and participants to establish trust, and “word of mouth” as affirmation of shared vision. Contextual barriers specific to rurality also impact partnership development capacity (limited staffing/resources) and logistics (travel distances). Findings suggest community centers serving older adults in rural communities are interested in collaborating with researchers to support cognitive health. However, logistical and capacity barriers limit the extent to which centers can commit to CBPR partnerships. Consideration of unique community contextual factors is necessary, requiring researchers to tailor partnership development to each center rather than adopting a “one size fits all” approach.

